# Determinants of Photodynamic Therapy Resistance in Cancer Cells

**DOI:** 10.3390/ijms252212069

**Published:** 2024-11-10

**Authors:** Alicja Dąbrowska, Jakub Mastalerz, Bartosz Wilczyński, Beata Osiecka, Anna Choromańska

**Affiliations:** 1 Faculty of Medicine, Wroclaw Medical University, Pasteura 1, 50-367 Wroclaw, Poland; alicja.dabrowska@student.umw.edu.pl (A.D.); jakub.mastalerz@student.umw.edu.pl (J.M.); bartosz.wilczynski@student.umw.edu.pl (B.W.); 2 Department of Clinical and Experimental Pathology, Wroclaw Medical University, T. Marcinkowskiego 1, 50-368 Wroclaw, Poland; beata.osiecka@umw.edu.pl; 3 Department of Molecular and Cellular Biology, Wroclaw Medical University, Borowska 211A, 50-556 Wroclaw, Poland

**Keywords:** photosensitizing agents, photochemotherapy, drug resistance

## Abstract

Photodynamic therapy (PDT) has emerged as a promising therapeutic approach owing to its non-invasive nature and minimal toxicity. PDT involves the administration of a photosensitizing agent (PS), which, upon light activation, induces a photodynamic reaction (PDR), leading to targeted cell destruction. However, developing resistance to PDT poses a significant challenge to its effectiveness. Various factors, including properties and administration of PSs, mediate this resistance. Despite the widespread use of substances like 5-aminolevulinic acid (5-ALA) and protoporphyrin, their efficacy is limited due to restricted tumor penetration and a lack of tumor targeting. To address these limitations, nano-delivery techniques and newer PSs like Aza-BODIPY and its derivatives, which offer enhanced tissue penetration, are being explored. In this paper, we provide an overview of resistance mechanisms in PDT and discuss novel methods, substances, and technologies to overcome resistance to improve clinical outcomes in tumor treatment.

## 1. Introduction

**Figure 1:**
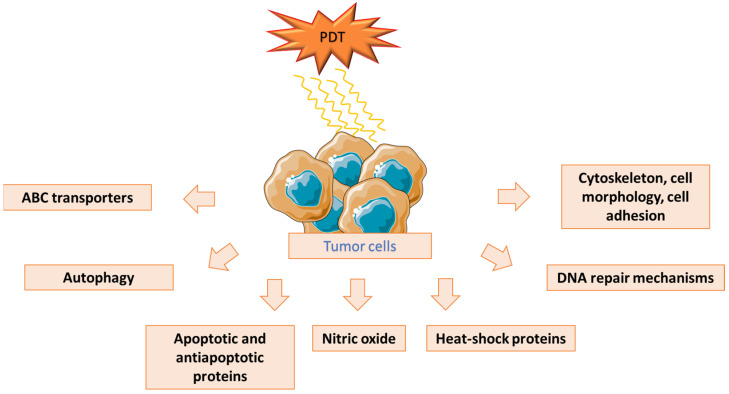
Mechanisms of cancer cell resistance to photodynamic therapy [1,2,3,4,5,6,9].

Photodynamic introduction therapy (PDT) is becoming a more and more popular therapeutic option. It is an evolving method used in oncological and non-oncological areas, as it is non-invasive and poses a low risk of toxicity. PDT entails the administration of a photosensitizing agent (PS), which later accumulates in target cells and absorbs the light in the phototherapeutic window (650–850 nm). As PSs use synthetic or natural chemical substances that transfer light energy to nearby molecules, they should only be toxic once light-activated, leading to a photodynamic reaction (PDR). Additionally, various factors could influence the positioning of the photosensitizer within the tumor, such as the photosensitizer’s charge, aggregation level, solubility (whether it is hydrophilic, hydrophobic, or amphiphilic), administration method, or the duration between administration and irradiation (referred to as DLI). Furthermore, the relevant light source is crucial in PDT since it must have an adequate wavelength and intensity for the PS. The light source may differ as long as the criteria needed are fulfilled. Intense visible, multispectral light from a lamp or a precise laser light (tuned to the PSs) are current effective light sources for PDT. Generally speaking, the activation of the PS leads to photochemical reactions, which are supposed to destroy the tumor cells without excessive normal tissue injury. The direct photodynamic effect leads to the interaction between reactive oxygen species (ROS) created in the PDR and various biological targets, which might disrupt homeostasis, changes in lipid metabolism, and ion transportation. ROS might also lead to the immune system’s response against tumor cells due to the activation of the protein kinase pathway, the expression of transcription factors and cytokines, and the release of inflammatory mediators, which may cause apoptosis and/or necrosis. Furthermore, ROS might disrupt the vascular walls and impair oxygen flow in the blood to the tumor microenvironment, including the neo-vasculature. Hypoxia of the cells might result in the necrosis of the cells, followed by the release of toxic metabolites to the tumor stroma [1,2,3]. Unfortunately, the development of resistance to PDT, mediated by various factors, including the properties and administration of PSs, poses a significant challenge to the effectiveness of this therapy. 5-aminolevulinic acid (5-ALA) is a widely used substance in PDT. Nevertheless, its therapeutic efficacy is limited due to restricted tumor penetration and targeting. There are numerous studies regarding nano-delivery techniques, which might decrease tumor resistance [4,5]. Another promising PS is Aza-BODIPY (Aza-Boron-dipyrromethene) and its derivatives. These compounds can be structurally modified to exhibit absorption and emission maxima in the red or near-infrared (NIR) spectral region, which enhances their suitability for deeper tissue penetration [6,7,8]. Another commonly used group of PSs is protoporphyrins due to their photosensitizing properties, although they also face limitations, such as lack of tumor targeting or low aqueous solubility [9]. The development of resistance to PDT is a severe problem; therefore, further research and new potential methods are crucial for enabling better results in tumor treatment. In our paper, we want to summarize and describe the mechanisms of the resistance to PDT (Figure 1), as well as new methods, substances, and technologies that might improve the clinical effects of PDT by decreasing its resistance [6].

For the literature search, we used PubMed, EMBASE, and Google Scholar databases, as well as references from relevant articles and internet sources. Search terms included “Photodynamic Therapy Resistance”, “PDT p-gp”, “PDT apoptosis”, “PDT ABC”, “PDT autophagy”, “PDT nitric oxide”, “PDT cytoskeleton”, “PDT heat-shock protein”, and “PDT DNA repair mechanisms”. We screened the titles and abstracts to identify relevant articles. Finally, we included 97 studies eligible for our review.

## 2. Mechanisms of PDT Resistance

### 2.1. ABC Transporters

#### 2.1.1. P-gp

The effect of antitumor medications, including PDT, depends on their concentration in tumor cells. Unfortunately, to defend themselves against treatment, many cancers produce proteins designed to remove chemotherapeutics outside the cell. Thus, the effective therapeutic concentration of the medication inside the cell and its cytotoxic effects are significantly reduced. These proteins belong to the ATP-binding cassette (ABC) superfamily transporter and are responsible for many therapeutic failures.

One of the most essential proteins is P-glycoprotein (P-gp), otherwise known as ABCB1, coded by the MDR1 gene. P-gp has an affinity to many chemically unrelated antineoplastic drugs, thereby evoking multidrug resistance. P-gp is a pattern recognition transporter with similarities to pattern recognition proteins [10]. The proposed action mechanism uses weak electrostatic interactions (hydrogen bonding, π-π stacking, π-cation interactions) to remove drugs through the lipid bilayer. Another hypothesis implies that it acts as a flippase [11].

**Figure 2:**
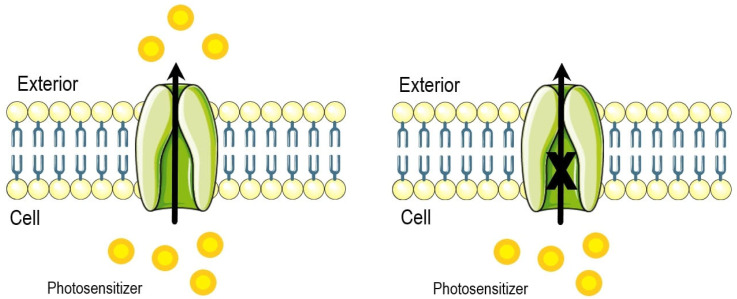
P-gp inhibitors, like verapamil, lead to an increased photosensitizer concentration inside the cell which contributes to enhanced photodamage [10,11,12].

A study investigated whether the use of verapamil can augment the phototoxic effects of PDT. Epithelial cells of the endometrium were collected from patients with cervical intraepithelial neoplasia and endometriosis. Before 5-aminolevulinic acid (ALA) therapy, epithelial cells were incubated in vitro with verapamil, the P-gp blocker (Figure 2). As an effect, cytotoxicity increased in both cell lines [12].

Resistance to PDT in breast cancer treatment remains a significant issue, and therefore, numerous studies regarding this area have been conducted. Recently, Aniogo et al. characterized resistant MCF-7 breast cancer cells developed by repeated cycles of PDT. They exposed wild-type MCF-7 cells to ZnPcS4 (a PDT drug) and analyzed the resistant population. They found that resistant cells showed a mesenchymal cell phenotype and enhanced expression of P-gp. Those results provide better insight into the development of new therapeutic options [13]. In a subsequent study, Aniogo et al. also used MCF-7 cells and ZnPcS4, but this time, overexpressed P-gp MCF-7 cells (MCF-7/DOX) were developed from wild-type MCF-7 cells with continuous exposure to different concentrations of doxorubicin. ZnPcS4 PDT had a compelling effect on MCF-7/DOX cells, becoming a promising treatment option for P-gp breast cancer. In addition, the sensitivity of MCF-7/DOX cells can be recovered after PDT treatment [14].

Another study compared the cytotoxic effects of PDT with the hexenyl ester of ALA (ALA-hx) between MCF-7 cells and adriamycin-resistant MCF-7(MCF-7/ADR) cells. Researchers found that, in MCF-7/ADR cells, intracellular production of ROS was more intense. This effect might result from the down-regulation of Nrf2-mediated antioxidant gene transcription and the up-regulation of protoporphyrin IX (PpIX) synthesis via the induction of coproporphyrinogen oxidase (CPO) and protoporphyrinogen oxidase (PPO). Furthermore, the expression of Nfr2-dependent antioxidant genes (γ-glutamylcysteine ligase, heme oxygenase-1, and quinone oxidoreductase) was down-regulated in MCF-7/ADR. Moreover, cells with Nf2 overexpression were less susceptible to ALA-hx PDT [15]

In the study from 2015, Wu et al. investigated the effects of the derivative of meta-tetra(hydroxyphenyl) chlorine (mTHPC) contained in pegylated liposomes as a photosensitizer on the expression of the MDR1 gene and P-gp in human nasopharyngeal carcinoma (NPC) cells. PDT with the previously mentioned photosensitizer induced overexpression of the MDR1 gene and P-gp protein production. It is worth mentioning that the PDT-mediated effect on cancer cells, also investigated in this study, was not diminished, while apparently, the photosensitizer used was not the P-gp substrate. This finding offers novel treatment options for MDR-resistant cells [16].

With the development of acridine-3,6-dialkyldithiourea hydrochlorides (AcrDTUs)—a new group of photosensitizers—efforts are being made to test their effectiveness in treating resistant cancer cells. Paulíková et al. assessed the efficacy of PDT/AcrDTUs in killing P-gp-overexpressing mouse leukemic cells L1210. The AcrDTUs appeared to be a valid option. However, using verapamil had no critical effect on propyl-AcrDTU (the most potent derivate) in terms of its accumulation [17].

#### 2.1.2. ABCG2

Another widely described ABC transporter is ABCG2, also known as breast cancer resistance protein (BCRP). ABCG2 can efflux a wide range of antineoplastic drugs, such as methotrexate, camptothecin-derived topoisomerase I inhibitors16, and photosensitizers. In normal tissues, ABCG2 protects cells from the devastating effects of phototoxic substances. Nevertheless, in cancer cells, it interferes with PDT. To overcome the resistance, different methods, e.g., ABCG2 inhibitors or the use of non-ABCG2 substrates, are investigated [18]. Pan et al. designed a study to investigate the resistance to methyl ester pyropheophorbide-a-mediated PDT (MPPa-PDT) in four different cell lines of human glioblastoma (U87, A172, SHG-44, and U251)—each line presented with various expressions of the ABCG2 protein. In the A172 line, the intracellular concentrations of MPPa-PDT were the lowest, while ABCG2 protein expression was the highest among all the lines [19].

Moreover, controlling ABCG2 expression may be a promising solution to increased PDT efficacy in cancers. In a study from 2015, two colon cancer cell lines, SW480 and HT29 cells, were exposed to treatment with a photosensitizer, pyropheophorbid-a (PPa). HT29 cells showed high ABCG2 expression, while SW480 showed the opposite. The higher uptake of PPa was observed in SW480 cells; nevertheless, after the pretreatment with ABCG2 and Ko-143, the uptake of PPa and, therefore, the efficacy of PDT in HT29 cells were substantially increased [20].

The potential of Ko-143 to overcome cancer cell resistance was also examined. Four human cell lines from the epidermis (HaCaT keratinocytes), esophagus (OE19 adenocarcinoma), brain (SH-SY5Y neuroblastoma), and bladder (HT1197 carcinoma) were incubated with ALA or MAL, with or without the Ko-143. It was proven that Ko-143 increased PDT efficacy in HaCaT, OE19, and HT1197 cells. However, this effect was not present in the SH-SY5Y cells [21]. A more recent study assessed the use of Ko-143 in the U251MG glioblastoma cells. ABCG2 expression in the U251MG cells was induced after doxycycline administration, and the cells with high production of the ABCG2 transporters were treated with ALA PDT. The use of the inhibitor photosensitizer accumulation and PDT efficiency was augmented [22]. Ishikawa et al. described kinase inhibitors, such as gefitinib, as a potential method to overcome PDT resistance. It was suggested that kinase inhibitors block Nrf2-mediated ABCG2 expression [23].

Lapatinib, a tyrosine kinase inhibitor used in breast and gastrointestinal cancer [24], was used to suppress ABCG2 efflux in human glioma cells treated with ALA-PDT. ABCG2 inhibition significantly increased the intracellular ALA metabolite concentration. In addition, lapatinib was compared to the intervention with iron chelator deferoxamine (DFO), and once more, the kinase inhibitor demonstrated greater efficacy [25]. Baglo Y. et al. described a way to overcome the ABCG2 and P-gp resistance using benzoporphyrin derivative (BDP) lipidation. BDP is a substrate of both P-gp and ABCG2. However, the phospholipid-conjugated BPD mitigates the resistance, thus increasing intracellular photosensitizer retention and enhancing PDT efficiency [26].

### 2.2. Apoptosis

**Figure 3:**
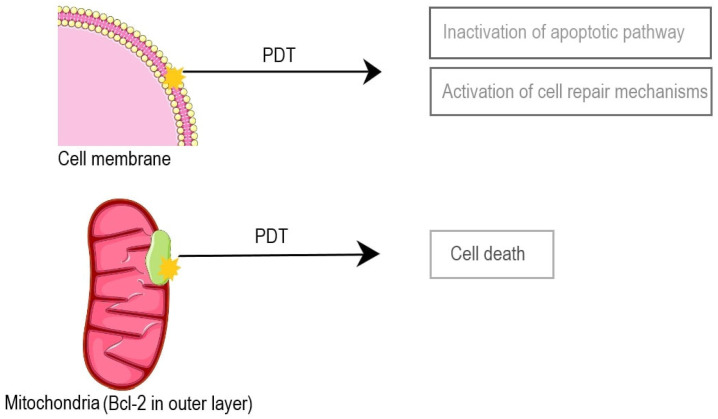
The targeted structure can determine PDT’s effect. PDT directed at anti-apoptotic proteins leads to cell death, yet damage to cell membranes evokes the opposite effect [27,28,29].

With the induction of apoptosis through various stimuli, signal transducing pathways are activated. It was demonstrated that PDT can up-regulate pathways coupled with Bcl-2 family members, caspases, and apoptosis-inducing factors, thereby promoting the process [27]. On the other hand, PDT, as a significant stressor, induces pathways designed to repair or tolerate the damage, which can lead to cell resistance [28]. The localization of the photosensitizer in the cell plays a crucial role in the mechanism of cell death. Depending on the nature of the photosensitizer used, whether hydrophobic or hydrophilic, they can have an affinity and inflict photodamage on the endoplasmic reticulum, Golgi apparatus, plasma membrane, and lysosomes. Damage to Bcl-2, anti-apoptotic proteins in the outer layer of mitochondria, leads to imminent cell death. However, damage to the plasma membrane may delay or stop the apoptotic pathway, simultaneously activating cell repair mechanisms. This phenomenon occurs when other cell structures, such as anti-apoptotic proteins, are targeted [29] (Figure 3).

Kessel et al. used two related photosensitizers in P388 murine leukemia cells to evaluate photodynamic responses to them. Tin thiopurine mediated damage to lysosomes and mitochondria, and tin octaethylpurpurin amidine (SnOPA) targeted lysosomes, mitochondria, and cell membranes. In the first case, the apoptotic nuclei were visible within 60 min of the PDT. However, apoptotic nuclei were absent in the second case until 24 h after PDT. Researchers provided evidence that SnOPA was relocated to the cytosol during irradiation, which resulted in the inactivation of enzymes necessary for the apoptotic process [30]. Later, it was found that these enzymes were predominantly procaspases 3 and 9 [31].

In a 2014 study, Kessel D and Reiners J investigated the low-dose lysosomal photodamage (lyso-PDT) potentiating the subsequent low-dose mitochondrial photodamage (mito-PDT). The N-aspartyl chlorin e6 with an affinity to lysosomes and the benzoporphyrin derivative (Bpd) targeting mitochondria were used, respectively, in murine hepatoma cells. It was found that Lyso-PDT with subsequent mito-PDT enhanced the cell death process. However, while reversing the sequence, the diminished effect was observed [32]. Two years later, Kessel proposed that this process involves a calpain-catalyzed cleavage of ATG5 to form a pro-apoptotic fragment. Ca^2+^ released from lysosomes was meant to be the initiating factor [33].

Photolone (Chlorin e6-PVP) is a water-soluble photosensitizer consisting of chlorine e6 (Ce6) molecules combined with polyvinylpyrrolidone (PVP). In murine colon carcinoma, CT-26 cells, Photolone localized itself mainly in mitochondria, lysosomes, Golgi apparatus, and around the nuclear envelope. In normal fibroblast cells (NHLC), it was found in minimal concentrations in the nucleus and no other subcellular organs. The photodamage to mitochondria and lysosomes of CT-26 cells was an essential factor in promoting apoptosis, judged by the formation of known apoptotic hallmarks [34].

### 2.3. Expression of Apoptotic and Anti-Apoptotic Proteins

The regulation of apoptosis is dependent on the expression of several anti-apoptotic proteins, such as Mcl-1, Bfl-1, Bcl-XL, Bcl-2, Bcl-w, and Bcl-B, and pro-apoptotic proteins, such as Bak, Bax, and Bok [35]. The activation or inactivation of these proteins can induce resistance to PDT. Granville D J et al. studied the effects of Bcl-2 overexpression on PDT resistance. Photosensitizer activation of benzoporphyrin derivative monoacid ring A (BPD-MA) induces procaspase-3 and -6 cleavage into active subunits. BPD-MA PDT on human acute myelogenous leukemia HL-60 cells with Bcl-2 overexpression inhibited caspase-3 and -6 activation, preventing apoptosis. This effect was more prominent after lower BPD-MA doses compared to higher doses [36].

Srivastava M. et al. investigated the involvement of Bcl-2 and Bax in PDT. Two approaches were implemented in the study. In the first approach, radiation-induced fibrosarcoma (RIF1) cells resistant to phthalocyanine (Pc4) were incubated with Bcl-2-antisense oligonucleotides. This action resulted in the down-regulation of the Bcl-2 protein, sensitization to Pc4-PDT, and increased apoptosis. However, the level of Bax remained unchanged. In the second approach, apoptosis-sensitive human epidermoid carcinoma (A431) cells were transfected with a eukaryotic expression vector (pSFFV/neo) containing an EcoRI fragment of human Bcl-2 cDNA, resulting in Bcl-2 overexpression. Interestingly, the PDT treatment in altered A431 caused increased apoptosis and up-regulation of the Bax protein. In both approaches, an increased Bax/Bcl-2 ratio enhanced PDT response [37].

A more recent study assessed the effects of the Bcl-2/Bcl-xL inhibitor ABT-263. Human glioblastoma cells and glioma stem-like cells were treated with 5-ALA PDT and ABT-263. Bcl-2/Bcl-xL inhibition enhanced cell apoptosis in combination with PDT. This result is probably related to the increased Noxa/Mcl-1 ratio, favoring the pro-apoptotic pathways [38]. Another BCL-2 inhibitor, ABT737, was integrated into upconversion zinc phthalocyanine (ZnPc) nanophotosensitizers. Due to the acidic pH of the cells, the solubility of these nanosystem particles changes after entering the lysosome, and ABT737 molecules are released, depleting the Bcl-2 levels. This leaves the tumor cells without the protection of the anti-apoptotic survival pathway [39]. Qiao L. et al. studied the effects of ALA-PDT on the proliferation and apoptosis of SCC cells A431 and COLO-16, focusing on the role of the JAK/STAT3 signal pathway. STAT3 and p-STAT3 protein levels were higher in cancer tissue compared to adjacent tissues. Moreover, the level of p-STAT3 in cancerous tissue correlates with tumor size and histopathological differentiation. ALA-PDT inhibited cell proliferation and promoted apoptosis, which was enhanced by STAT3 knockdown. ALA-PDT also weakened the expression of STAT3’s target genes Bcl-2 and Bax [40].

### 2.4. Autophagy

Autophagy is a process of cell self-degradation, which is vital for balancing energy sources during stress or development. It plays an essential role in tumor progression and the response to antineoplastic therapies. Autophagy is a double-edged sword in PDT. Photodamage can induce autophagy, leading to cancer cell death. However, cancer cells can use autophagy to promote their growth and induce resistance to PDT. In a 2021 study, the efficacy of Ce6-PDT in the treatment of human colorectal cancer and the role of autophagy in Ce6-PDT were evaluated. Ce6-PDT was used on SW480 cells with or without the autophagy inhibitor 3-methyladenine (3MA). Ce6-PDT induced apoptosis via the mitochondrial pathway and autophagy in SW480 cells. What is more, the use of 3MA potentiated the cell response to Ce6-PDT, increasing the rate of apoptosis [41]. Another study also assessed the effects of Ce6-PDT in colon cancer cells, this time combined with oxaliplatin (L-OHP). After long-term use, L-OHP increased the rate of autophagy and the expression of ABCB1 and ATG5 proteins. It was stated that autophagy plays a protective role against Ce6-PDT and L-OHP [42]. In Ce6-PDT, autophagy and apoptosis depend on ROS production, as shown in a study on SW620 colon cancer cells. ROS has been reported to activate the p38MAPK pathway, probably preventing photodamage, as it leads to downstream signaling of ROS. The inhibition of the p38MPAK pathway with siRNA or the inhibitor SB203580 enhanced autophagy and apoptosis. Moreover, when autophagy inhibitor 3-methyladenine/Bafilomycin A1 was used, PDT efficacy was significantly reduced [43].

As mentioned before, depending on the context, autophagy in PDT promotes cell survival or death. A group of researchers tried to determine the role of autophagy in Photofrin-based PDT. Its use was associated with robust autophagy induction in both examined cancer cell lines, Hela and MCF-7, leading to decreased cytotoxicity. The down-regulation of AGT5, one of the critical genes in autophagosome formation, only partially blocked the autophagic response, and the efficacy of PDT was only minimally improved. However, ATG5 knockout in the Hela cell line with the application of CRISPR/Cas9 genome editing significantly increased the phototoxicity through an enhanced apoptotic response and more significant accumulation of carbonylated proteins [44].

Polyhylipoprotein (PLP) is a recently introduced nanoparticle that has high tumor selectivity and biocompatibility. What is more, it demonstrated its usefulness as a photosensitizer in PDT. It was established that PLP utilizes previously unknown PDT mechanisms concentrating on autophagy. The dynamics of phagosome formation were different between healthy cells and cancer cells. In RGK1 cancer cells, the phagosome size was more significant than that of RGM 1 healthy cells. After photosensitization in RGK 1 cells, the phagosome ultimately ruptured, inducing cytotoxicity. However, in RGM 1 cells, the phagosomes were not destroyed after PDT [45].

### 2.5. The Role of Nitric Oxide

**Figure 4:**
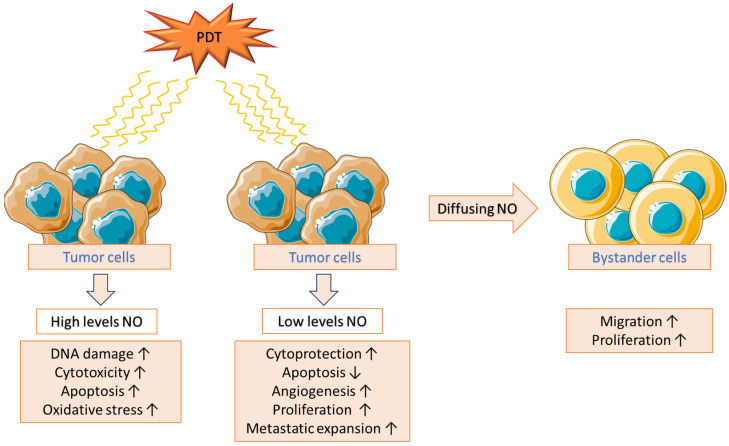
The effect of nitric oxide produced as a result of photodynamic therapy (PDT) on cancer cells, including bystander cells [47,48,49,50,51,52].

Nitric oxide is an unstable and bioactive inorganic chemical compound with nitrogen in the second oxidation state. Due to its role in many physiological processes and its connection with the pathogenesis of many pathologies, it has fascinated scientists for several decades [46]. The nitric oxide synthase (NOS) family is responsible for its production, including three isoforms: neuronal (nNOS/NOS1), inducible (iNOS/NOS2), and endothelial (eNOS/NOS3) [47]. In the context of a response to PDT, nitric oxide plays a dual role. Low levels of NO induced by PTD exert cytoprotective effects due to the activation of the pro-survival NF-κB/Snail/YY1/RKIP loop as well as inhibition of the anti-survival RKIP. On the other hand, high levels of NO induced by PDT have antitumor cytotoxic effects, which are the result of the inhibition of NF-κB, Snail, and YY1 and the activation of RKIP [48]. Moreover, NO, in addition to negatively affecting the effectiveness of PDT, can also stimulate the proliferation and aggressive migration of cancer cells [49] (Figure 4). Visible light exposure of 5-aminolevulinic acid-mediated U87 or U251 human glioblastoma cells led to a three- to fourfold increase in iNOS levels. It did not cause significant changes in nNOS levels. iNOS induction contributed to an increase in intracellular NO secretion. Furthermore, the use of NO scavengers and iNOS inhibitors was associated with an increase in apoptosis induced by ALA/light treatment. Surviving cells showed more significant proliferation and migration than cells from the control group [50,51]. Later, the same group showed that the NF-κB-mediated iNOS response was associated with increased epigenetic levels of Brd4 and acK310 in the p65 subunit of NF-κB. Moreover, using the Brd4 inhibitor, JQ1 suppressed iNOS expression and NO production. This type of therapy reduced the aggressiveness of cancer cells more effectively than the use of iNOS inhibitors. This discovery may contribute to a significant improvement in the effectiveness of PDT [52]. iNOS/NO was also induced in MDA-MB-231 human breast cancer cells in vitro and in vivo which were sensitized with ALA and then irradiated. iNOS inhibitors (GW274150, 1400 W) and NO traps (cPTIO) promoted PDT-induced apoptosis. The PDT-mediated up-regulation of pro-survival proteins such as Bcl-xL, survivin, and S100A4 was also observed and was inhibited by 1400 W [53].

Similar to breast cancer and glioblastoma cells, iNOS/NO induction was also demonstrated in human prostate cancer PC3 cells subjected to PDT. Furthermore, ALA/light treatment-dependent up-regulation of extracellular matrix metalloproteinase-9 (MMP-9) and α6 and β1 integrins and down-regulation of the MMP-9 inhibitor TIMP-1 were observed. This effect was strongly inhibited after the use of an iNOS inhibitor [53]. A pro-tumorigenic effect of endogenous nitric oxide on bystander cells was also demonstrated, conditioned by NO diffusion from tumor cells that survived PDT (Figure 4). ALA-PDT-targeted tumor cells were separated from untargeted cells in a culture dish using impermeable silicone rings and irradiated with LED. After treatment and removal of the silicone ring, increased migration and proliferation of bystander cells were observed. Moreover, this effect correlated with the level of iNOS/NO induction, which was highest in PC3 cells. In an actual tumor, such an effect could, in unfavorable cases, cause the spread of malignant tumor cells [54,55].

### 2.6. The Role of Heat-Shock Proteins

Reactive oxygen species induced by PDT lead to the activation of various protective mechanisms, including the expression of heat-shock proteins (HSPs). Intracellular HSPs are associated with anti-apoptotic function. In turn, extracellular HSPs and membrane-associated HSPs are thought to mediate immune functions [56]. It has been observed that HSP27, HSP34, HSP60, HSP70, HSP90, HSP110, GRP glucose-regulated proteins (GRP74, GRP78, and GRP100), and HO-1 are involved in the response to PDT [57]. In a study using two breast cancer cell lines, one of which (DB46) was characterized by high constitutive levels of HSP27 and the other (DC4) by normally low levels of HSP27, it was observed that the LD50 values for PDT were higher for the cell line characterized by higher levels of HSP27. Moreover, based on TUNEL assays and fluorescence microscopy, it was found that PDT resistance involved both necrosis and apoptosis limitation in DB46 cells [58]. PDT was also shown to promote HSP27 expression in colon cancer cells, leading to increased autophagic cell survival and reduced apoptosis of cancer cells. Furthermore, PDT-resistant skin cancer cells overexpressed the HSP27/autophagy axis [59]. On the contrary, it has been shown that down-regulating HSP27 in human oral cancer KB cells leads to the attenuation of PDT-induced apoptosis through the inhibition of apoptotic proteins. It was also observed that silencing HSP27 affected the expression of Bax, Bcl-2, and PARP proteins in PDT-induced cells [60]. The same group also observed PDT-induced decreased HSP27 expression and increased HSP70 expression in head and neck cancer cells. Moreover, treatment of these cells with LC3II and PARP-1 inhibitors led to an increased expression of both HSP27 and HSP70. Down-regulation of HSP27 and HSP70 also contributed to developing resistance to PDT through autophagy and apoptosis [61]. Significant overexpression of mitochondrial HSPA9 and HSP60 mRNAs has recently been demonstrated in tumor tissue and in MB49 bladder cancer cell cultures [62]. Currently, functional nanomaterials are promising for breaking PDT resistance. Small HSP inhibitors that competitively bind to the ATP-binding site of HSPs can be used for this purpose. Nanomaterials can also deliver glucose deprivation agents (such as glucose oxidase) to tumor cells, thereby reducing ATP and, consequently, HSP production. What is more, co-administration of a small-molecule HSP inhibitor and an anticancer drug could enable the combination of chemotherapy and PDT [63].

### 2.7. DNA Repair Mechanisms

**Figure 5:**
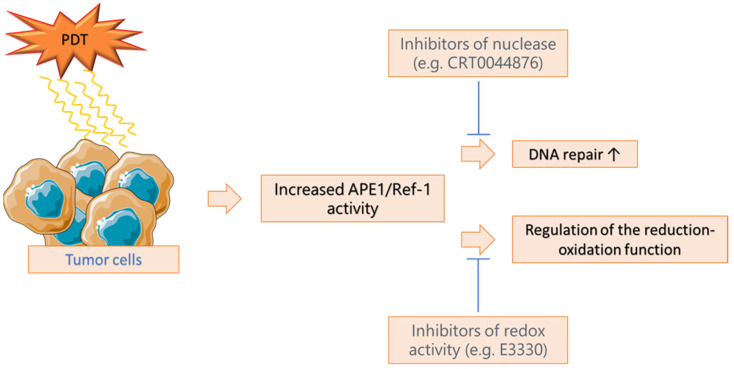
The dual role of apurinic/apyrimidinic endonuclease 1/redox effector factor 1 (APE1/Ref-1) in the development of resistance to PDT [64,65,66,67].

The therapeutic effect of PDT can be compensated by mainly activating DNA repair mechanisms in the cell. This aspect still needs to be thoroughly understood, as we have yet to learn all the repair mechanisms induced by PDT [64]. It has been shown that in U87 glioma cells resistant to Photofrin-mediated PDT (Ph-PDT), Alpha-Ketoglutarate Dependent Dioxygenase Homolog 2 (ALKBH2) is involved in reversing Ph-PDT-induced DNA damage. A decreased expression of ALKBH2 led to increased apoptosis. Moreover, the development of such resistance was mediated by the tumor protein TP53, which is bound to the ALKBH2 promoter [65]. Based on the analysis of isolated PDT-resistant glioblastoma multiforme cells, it was shown that the resistance of these cells was due to a number of mechanisms, among which are the efficient repair of oxidative DNA damage and the repair of DNA breaks. Furthermore, higher apurinic/apyrimidinic endonuclease 1 (APE1) activity and the increased expression and activation of DNA damage kinase were observed in resistant glioblastoma multiforme cells from the U-87 MGR line. In the future, they may become good targets for sensitizing cells to PDT treatment [66]. Apurinic/apyrimidinic endonuclease 1/redox factor-1 (APE1/Ref-1) regulates the reductive–oxidative function and DNA repair function in response to oxidative stress, making it a protein associated with poor prognosis (Figure 5). Various APE1/Ref-1 inhibitors that can inhibit reductive–oxidative function (e.g., E3330) and DNA repair function (CRT0044876) can overcome cellular resistance to therapy. Excellent sensitization effects were obtained using PDT and E3330 against HeLa cervical cancer cells [67,68]. It was also previously shown that APE1 silencing mediated by the Ad5/F35-shAPE1 adenoviral vector reduced APE1 protein expression in cultured cells of the A549 non-small-cell lung cancer cell line and their xenografts from nude mice. This reduced the DNA repair capacity for single-strand breaks and decreased the activation of certain stress-related transcription factors, such as hypoxia-induced factor (HIF)-1, resulting in more significant apoptosis of PDT-treated cancer cells [69]. Recently, it has been shown that PDT-resistant vulvar cancer cells increased APE1 activity in response to irradiation and accumulated and excreted less porphyrins than PDT-sensitive cells. Moreover, APE1 inhibition was associated with a significant improvement in the reaction of PDT-resistant cells [70]. It was observed that chlorine 6 (Ce6)-induced PDT led to the elevation of ROS in Lewis lung cancer cells, which resulted in the occurrence of DNA double-strand breaks (DDSBs) and an increased expression of DDR γ-H2A.X, p-ATM, and p53 proteins. However, the degree of cell apoptosis was relatively low due to ATM-related DDR. Moreover, this effect could be reversed by using an ATM inhibitor [71].

DNA repair induced by PDT-induced damage can be reduced by inhibiting various enzymes involved in repair processes. For example, chlorine e6 and the PARP inhibitor olaparib (Ola) can be combined. CeOla can inhibit PARP activation, up-regulate γ-H2AX, and reduce Rad51 expression [72]. Recently, a hyaluronic acid (HA)–Ce6–olaparib (Ola) micelle (laHCCO) has also been developed that combines the effects of HA on cancer stem cells (CSCs) and the inhibition of PDT-induced DNA damage repair by Ola [73]. MTH1 is also a known enzyme that is involved in developing resistance by participating in the DNA repair process. Recently, a hypoxia-activated FTPA nanosystem system has been proposed to increase the sensitivity of cancer cells to PDT by releasing the MTH1 activity inhibitor TH588 [74].

### 2.8. Cytoskeleton, Cell Adhesion, and Cell Morphology

The cytoskeleton is an extraordinarily complex and structured network consisting of actin filaments, intermediate filaments, and microtubules. It involves numerous physiological processes, and its disorders play an essential role in developing many diseases [75]. We know that one of the targets of PDT is the cytoskeleton [76]. It was also observed that photodamage to mitochondria in NIH3T3 (mouse embryonic fibroblast) after ALA-PDT correlated with changes in cell structure and increased cell adhesion [77]. It has been shown that changes in cell shape, cell adhesion properties, and the cytoskeleton can affect the process of metastasis formation and the resistance of cancer cells to PDT. Based on the analysis of microfilament disorganization after ALA-PDT treatment on the normal breast cell line HB4a and its tumorigenic and resistant counterpart HB4a-Ras, it was suggested that F-actin and other components of the cytoskeleton may influence the development of resistance to PDT [78]. Recently, it has also been observed that HB4a-Ras cells show abnormal organization of actin filaments and lower levels of E-cadherins and β1 integrins compared to cells of the parental line. Moreover, an increase in PDT resistance has been demonstrated in free-floating cell groups compared to the adherent cells, which have a similar ability to Ras in increasing cell survival under conditions where cell–extracellular matrix interactions are limited [79]. Furthermore, analysis of PDT-resistant SCC-13 squamous cell carcinoma cells revealed a more fibroblastic structure, a greater wound closure capacity, and a higher expression of cell–substrate adhesion proteins than cells from the parental line [80]. Also, resistant lines derived from LM3 mammary adenocarcinoma showed a loss of stress fibers and abnormal organization of the actin cortex envelope. However, no changes in the expression of actin, E-cadherin, vinculin, or beta-catenin were observed [81]. It was recently observed that in vulvar cancer cells, PDT can induce changes in the cytoskeleton that can lead to epithelial–mesenchymal transition (EMT) or increased adhesion. In turn, EMT and cell–cell adhesion may contribute to the activation of various anti-apoptotic pathways. Moreover, we observed that in the A-431 line, the development of resistance was associated with increased levels of β-tubulin and E-cadherin. At the same time, PDT induced significant changes in their levels only in sensitive cells. This could mean that increased levels of β-tubulin and E-cadherin may be associated with increased cell adhesion and resistance to PDT [82].

## 3. Recent Developments in PDT and Its Application Against Multidrug-Resistant Cancers

MDR, which stands for multidrug resistance, plays a crucial role in the development of chemotherapeutic drug resistance in various malignancies. It significantly contributes to the reduced efficacy of chemotherapy across a spectrum of cancers, such as breast cancer, lung cancer, blood malignancies, oral cancer, and others. Numerous recent studies have researched the use of PDT to decrease the effects of MDR on the treatment. Recently, researchers have used a nanoparticle-mediated photodynamic therapy (NPs/PDT) approach to overcome both types of MDR in lung cancer. They used a photosensitizer, 5,10,15,20-Tetrakis(4-hydroxy-phenyl)-21H,23H-porphine (pTHPP), which was loaded into poly(D, L-lactide-co-glycolide) (PLGA)–lipid hybrid nanoparticles, and then analyzed the results from two different A549 human lung adenocarcinoma cell lines. In the drug-selected MDR model, A549RT-eto cells displayed a 17.4-fold resistance to Etoposide and a 1.8-fold resistance to Paclitaxel in comparison to the A549 parental cells. In the metastasis-associated MDR model, floating A549 cells showed resistance to Etoposide (11.6-fold) and Paclitaxel (57.8-fold) compared to attached A549 cells. Interestingly, the floating cells did not resist the photocytotoxic effect of the NPs/PDT. Thus, this study suggests that PLGA–lipid hybrid nanoparticles show promise in delivering photosensitizers or chemotherapeutic drugs for treating both drug-selected and metastasis-associated MDR lung cancer cells [83]. Additionally, the versatile plasmonic CuO/Cu_2_O truncated nanocube-based theranostic nanomedicine, which sensitizes the formation of singlet oxygen (1O2) to exert nanomaterial-mediated photodynamic therapeutics (NIR-II NmPDT), and absorbs long NIR light (i.e., 1550 nm) in the biological window III (1500–1700 nm) to exert nanomaterial-mediated photothermal therapeutics (NIR-III NmPTT), seems to effectively destroy multidrug-resistant lung tumors. Therefore, H69AR lung cancer cells did not develop resistance to plasmonic CuO/Cu_2_O TNC-based nanomedicines [84]. Moreover, due to the increasing importance of synergistic therapies, various new delivery systems encapsulated with dual agents are explored. Busa P et al. evaluated the efficacy of (IBN)-1-based mesoporous silica nanoparticles (MSNs) for multifunctional drug delivery in overcoming drug resistance. These hybrid nanocomposites were found to be effectively internalized through endocytosis and proficiently transported curcumin (Cur) and cisplatin molecules embedded in them into the cytosol. Additionally, using Cur photosensitizers within the nanochannels of MSNs led to heightened cellular reactive oxygen species (ROS) levels when exposed to light irradiation. Consequently, IBN-1-Cur-CP demonstrated outstanding efficacy in anticancer therapy against MES-SA/DX5-resistant cancer cells, attributable to the synergistic effects of both chemo- and photodynamic treatments [85]. Furthermore, a tumor-targeting nanoplatform was developed in another study to deliver mitochondria precisely—and endoplasmic reticulum (ER)-targeting PDT agents—to desired sites for dual organelle-targeted PDT. Therefore, mitochondrial dysfunction leads to diminished ATP production, contributing to the reversal of multidrug resistance (MDR). This nanoplatform, characterized by its near-infrared (NIR) responsive attributes and capacity to target both tumors and subcellular organelles, presents a promising approach for successful MDR cancer therapy [86].

Another encouraging study confirming the potential of nanoparticles as therapeutic methods is the research carried out by Shi X et al. They found that novel redox-responsive, chondroitin sulfate-based nanoparticles might improve the chemo-photodynamic therapy of breast cancer due to the down-regulation of the p-gp on MCF-7/ADR cells and thereby improve the anticancer efficacy of PTX against MCF-7/ADR cells. Furthermore, the novel nanoplatform exhibited effective in vivo MDR inhibition and anti-metastasis efficacy, creating further therapeutic possibilities [87]. Rezaeivala et al. created CoFe_2_O_4_ theranostic magnetic nanoparticles coated with spiky gold nanoparticles and assessed their photothermal effects combined with the photodynamic and chemotherapeutic effects of mitoxantrone (MTX) under in vitro conditions in the aggressive breast cancer cell line MDA-MB-231. The enhanced cell death was found to be due to the convergence of the light source’s emission spectrum with the nanostructure’s absorption spectrum [88].

To overcome the severe consequences of MDR in gastric cancer treatment, Li H et al. evaluated the results of the self-enhanced Mn_3_O_4_ nanoplatforms (MPG NPs), which could react with glutathione to produce Mn^2+^ and thus mediate in vivo real-time MDR monitoring. It turned out that MPG NPs exhibit hemodynamic activity, enabling the conversion of endogenous hydrogen peroxide within tumor cells into a highly toxic hydroxyl radical through a Fenton-like reaction under acidic pH conditions, thus serving as a form of hemodynamic therapy. Notably, the presence of Mn_3_O_4_ significantly amplifies the effectiveness of chemodynamic treatment due to its great photothermal conversion capabilities [89].^,^ Recently, a multi-therapeutic nanotool was designed by merging the photothermal properties of gold nanorods (AuNRs) with the photodynamic activity of the photosensitizer verteporfin to research potential therapeutic methods to treat colon cancer. They used human colon cancer cell line (HCT116) murine xenograft models to assess whether AuNRs can prevent tumor growth. The final results suggest that combining PTT and PDT might lead to xenograft depletion [90].

Likewise, PSs are characterized by high selectivity, minimizing resistance to cancer treatment. Combining PTT and PDT may offer a viable approach to enhance their anticancer effectiveness. At the same time, it generates elevated oxidative stress in mitochondria, potentially causing damage to proteins, lipids, and DNA. In PT/PD combination modalities, the lipophilic cationic material disrupts electron transport chains and induces damage to the mitochondrial membrane [91]. Targeting mitochondria in cancer therapy might lead to eliminating tumor cells and can be used to overcome the MDR issue through numerous methods. Lv et al. designed hollow copper sulfide polydopamine-coated nanoparticles as photothermal agents and heat-sensitive carriers for TH302, a hypoxia-activated prodrug. Ce6 and TPP were attached to the nanoparticle surfaces to specifically target mitochondria, ensuring the activity of chemotherapy drugs and preventing drug resistance. By employing dual laser radiation at 660 and 808 nm, this study demonstrated an increased production of reactive oxygen species (ROS) and an enhanced release of TH302 in tumor cells [92]. In addition, PDT seems to be an effective treatment for radiation-resistant tumors since it might be used in recurrent esophageal cancer patients after radiation chemotherapy. There still is not enough research to explain why PDT is effective against radioresistant cancers; nevertheless, one study suggests that it is due to X-ray-resistant cells’ high production of mitochondrial-derived ROS. Consequently, greater amounts of ROS cause improved nuclear translocation of NF-κB, which results in higher NO production, while the expression of PEPT1, enabling the import of 5-ALA, is up-regulated [93].

## 4. Conclusions

PDT is gaining increasing popularity as a therapeutic approach. It is used in oncological and non-oncological fields due to its non-invasive nature and minimal risk of toxicity, making it an evolving treatment method. Unfortunately, the growing issue of resistance to PDT poses a significant challenge. This resistance is influenced by several factors, notably the role of ABC transporters, such as p-gp, which reduce the final concentration of photosensitizers (PSs), potentially leading to decreased treatment efficacy. This is why targeting these transporters, especially ABCG2, seems to be a potential treatment method, but further research is needed. What is more, apoptosis regulation might significantly impact the course of PDT. Evidence suggests that inhibition of anti-apoptotic proteins, like Bcl-2 and Bcl-xL, can amplify apoptosis, thus potentially improving treatment outcomes. Similarly, autophagy’s dual role—as both a survival and death mechanism—requires careful modulation to optimize PDT efficacy, particularly in treatments involving Ce6-PDT and LOHP. Moreover, further studies regarding the role of NO and HSPs should be considered, as they might be used in different treatment strategies to improve the results. PDT is also considered to improve the treatment results in MDR cancers. PDT’s potential in treating multidrug-resistant (MDR) cancers is encouraging, especially as it can down-regulate critical resistance factors like p-gp in resistant cancer cells. Yet, while findings are promising, further research is crucial to optimize PDT protocols, refine target mechanisms, and extend clinical applications. Furthermore, developing more sophisticated PDT treatment methods accounting for specific tumor characteristics may result in more consistent, favorable outcomes across a wider range of cancer types.
